# Multi-Segment TFT Compact Model for THz Applications

**DOI:** 10.3390/nano12050765

**Published:** 2022-02-24

**Authors:** Xueqing Liu, Trond Ytterdal, Michael Shur

**Affiliations:** 1 Department of Electrical, Computer and Systems Engineering, Rensselaer Polytechnic Institute, Troy, NY 12180, USA; shurm@rpi.edu; 2 Department of Electronic Systems, Norwegian University of Science and Technology, 7491 Trondheim, Norway; trond.ytterdal@ntnu.no; 3 Electronics of the Future, Inc., Vienna, VA 22181, USA

**Keywords:** terahertz, TFT, compact model, SPICE

## Abstract

We present an update of the Rensselaer Polytechnic Institute (RPI) thin-film transistor (TFT) compact model. The updated model implemented in Simulation Program with Integrated Circuit Emphasis (SPICE) accounts for the gate voltage-dependent channel layer thickness, enables the accurate description of the direct current (DC) characteristics, and uses channel segmentation to allow for terahertz (THz) frequency simulations. The model introduces two subthreshold ideality factors to describe the control of the gate voltage on the channel layer and its effect on the drain-to-source current and the channel capacitance. The calculated field distribution in the channel is used to evaluate the channel segment parameters including the segment impedance, kinetic inductance, and gate-to-segment capacitances. Our approach reproduces the conventional RPI TFT model at low frequencies, fits the measured current–voltage characteristics with sufficient accuracy, and extends the RPI TFT model applications into the THz frequency range. Our calculations show that a single TFT or complementary TFTs could efficiently detect the sub-terahertz and terahertz radiation.

## 1. Introduction

Thin-film transistor (TFT) technology has found numerous applications including large area and flexible displays [1,2], sensitive skin [3], biomedical and chemical sensors [4], and radio frequency identification (RFID) sensors [5]. The TFT liquid-crystal display (LCD) attained approximately USD 164 billion market size in 2020 and is expected to grow with a 5.2% rate for 2021–2026 [6]. The emergence of novel TFT materials such as ZnO [7,8], InGaZnO [9], carbon nanotube (CNT) [10], and organic materials [11] has been improving the properties of TFTs including the carrier mobility, current-carrying capacities, stability, and mechanical flexibility. The progress in scaling down TFT sizes has resulted in the design and fabrication of high performance TFTs [12,13,14]. All these efforts have shrunk the performance gap between TFT and complementary metal-oxide-semiconductor (CMOS) technologies and allowed for emerging higher frequency applications (even into the THz range) as shown in this paper. Supporting these applications requires the development of advanced compact models for TFTs to complement the numerical simulations [15,16,17]. The conventional RPI TFT model does not account for the dependence of the channel layer thickness on the gate voltage and is not valid at very high frequencies such as the THz range. In this work, we present an improved compact model based on the RPI TFT model [18] and the Automatic Integrated Circuit Modeling SPICE (AIM-SPICE) MOSA1 platform [18,19] but accounts for a non-exponential slope in the subthreshold regime (varying subthreshold slope), non-trivial capacitance dependence on the gate bias, and accommodates the inclusion of the parasitics related to the gate impedance. Even more importantly, the model accounts for non-local potential distribution in the device channel by using a multi-segment (nonlinear transmission line) approach that has been successfully used for the THz SPICE Si CMOS model implementation [20,21]. These novel features enable an excellent fitting with the measured DC characteristics for TFTs and significantly extend the application frequency range of the TFT model.

Section 2 presents the equivalent circuit and the basic equations of the new multi-segment TFT unified charge control model (UCCM). Section 3 presents the simulation results of the long channel and short channel TFTs including the DC characteristics, distributed impedance, and cutoff frequency, and analyzes the THz response of oxide short channel TFTs. Section 4 summarizes the modeling results and provides suggestions for further studies.

## 2. Model Details

**Figure 1:**
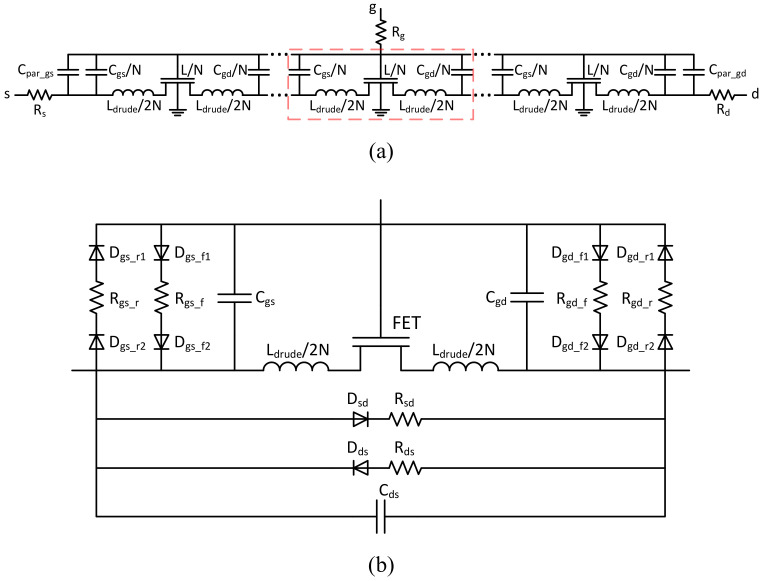
Equivalent circuit of the multi-segment SPICE model for TFT (**a**) and equivalent circuit for each segment including leakage components (**b**). Reprinted with permission from ref. [21]. Copyright 2018 IEEE Transactions on Electron Devices.

Figure 1 shows the equivalent circuit of the TFT SPICE model with channel segmentation and the equivalent circuit for each segment including leakage components [21,22]. The multi-segment SPICE model is based on the UCCM, which calculates the distributed nonlinear impedances of the intrinsic FETs and the nonlinear internal capacitances.

The equations for the TFT SPICE model are described by the UCCM [18,19] where the unified drain-to-source current of the intrinsic TFTs given by

(1) $$I_{ds}=\frac{g_{chi}V_{ds}\left(1+\lambda V_{ds}\right)}{{\left[1+{\left(V_{ds}/V_{sate}\right)}^{m}\right]}^{1/m}},$$

$V_{ds}$ is the extrinsic drain-to-source voltage, λ is an empirical factor related to the gate length modulation, *m* is a parameter determining the knee region shape of the output characteristics, $V_{sate}=I_{sat}/g_{chi}$ is the effective extrinsic saturation voltage, $g_{chi}$ is the intrinsic linear channel conductance, $I_{sat}$ is the drain saturation current,

(2) $$I_{sat}=\frac{g_{chi}V_{gte}}{1+\sqrt{1+{\left(V_{gte}/V_{L}\right)}^{2}}},$$

(3) $$g_{chi}=\frac{qn_{s}\mu W}{L},$$

(4) $$V_{gte}=\eta V_{th}\left[1+\frac{V_{gt}}{2\eta V_{th}}+\sqrt{\delta^{2}+{\left(\frac{V_{gt}}{2\eta V_{th}}-1\right)}^{2}}\right],$$

(5) $$\mu =\mu_{0}\frac{\alpha V_{gte}^{\gamma }}{1+\alpha V_{gte}^{\gamma }},$$

(6) $$\eta =\eta_{2}+\frac{\eta_{2}-\eta_{1}}{1+\exp(\frac{V_{gst}-V_{gs}}{\Delta V_{gst}})},$$

$V_{gte}$ is the effective gate voltage swing, μ is the gate voltage dependent electron mobility, $\mu_{0}$ is the low field mobility, α and γ are fitting parameters determining the gate voltage dependence for the mobility, η is the subthreshold ideality factor, $\eta_{1}$ is the ideality factor in the moderate subthreshold region, $\eta_{2}$ is the ideality factor in the deep subthreshold region, $V_{gst}$ is the voltage between the two subthreshold regions, $\Delta V_{gst}$ is the parameter determining the width of the transition, *q* is the electric charge, *W* is the gate width, *L* is the gate length, $n_{s}=n_{s0}/{\left[1+{\left(n_{s0}/n_{max}\right)}^{\kappa }\right]}^{1/\kappa }$ is the channel electron sheet density, $n_{max}$ is the maximum electron sheet density in the channel, κ is a characteristic parameter for the transition to saturation in $n_{s}$, $n_{s0}=n_{0}\ln(1+\exp\left(V_{gt}/\left(\eta V_{th}\right))\right)$ is the ideal unified electron sheet density, $n_{0}=\epsilon_{i}\eta V_{th}/\left(q\left(d_{i}+\alpha_{g}V_{gt}\right)\right)$ is the gate voltage dependent electron sheet density at threshold, $\epsilon_{i}$ is the dielectric layer permittivity, $V_{th}$ is the thermal voltage, $d_{i}$ is the dielectric layer thickness, $\alpha_{g}$ is a parameter determining the gate voltage dependence for the electron sheet density, $V_{gt}=V_{gs}-V_{T}+\sigma V_{ds}$ is the gate voltage swing, σ is the drain-induced barrier lowering (DIBL) parameter, $V_{gs}$ is the extrinsic gate-to-source voltage, $V_{T}$ is the threshold voltage, $V_{L}=v_{s}L/\mu$, $v_{s}$ is the saturation velocity, δ is a parameter determining the width of the transition from the above to subthreshold region.

The nonlinear capacitances for the TFT compact model are given by

(7) $$C_{gs}=\frac{2}{3}C_{gc}\left[1-{\left(\frac{V_{gte}-V_{dse}}{2V_{gte}-V_{dse}}\right)}^{2}\right],$$

(8) $$C_{gd}=\frac{2}{3}C_{gc}\left[1-{\left(\frac{V_{gte}}{2V_{gte}-V_{dse}}\right)}^{2}\right],$$

(9) $$C_{gc}=\frac{\epsilon_{i}LW\exp\left(\frac{V_{gt}}{\eta_{c}V_{th}}\right)}{\left(d_{i}+\alpha_{g}V_{gt}\right)\left[1+\exp(\frac{V_{gt}}{\eta_{c}V_{th}})\right]},$$

(10) $$V_{dse}=\frac{1}{2}\left[V_{ds}+V_{gte}-\sqrt{{\left(\eta_{c}V_{th}\delta_{c}\right)}^{2}+{\left(V_{ds}-V_{gte}\right)}^{2}}\right],$$

$C_{gc}$ is the differential gate-to-channel capacitance incorporating both the above and the subthreshold regimes, $\eta_{c}=\eta \left(1+\beta V_{gte}\right)$, β is a parameter determining the gate voltage dependence for the subthreshold ideality factor for the capacitance, $\delta_{c}$ is the parameter determining the width of the transition from the above to subthreshold region for the capacitance, $V_{dse}$ is the effective extrinsic drain-to-source voltage.

The SPICE model includes the kinetic or Drude inductances $L_{drude}=\tau R_{ch}$, where $R_{ch}$ is the channel resistance, τ=mμ/q is the electron momentum relaxation time, *m* is the electron effective mass [23]. The model also accounts for the leakage components, the series resistances, and the parasitic capacitances [21,22] shown in Figure 1. The model can be implemented in SPICE by implementing the above equations in the Verilog-A language and could be used in the circuit simulators such as Cadence and ADS.

## 3. Results and Discussions

### 3.1. DC Current-Voltage Characteristics

**Figure 2:**
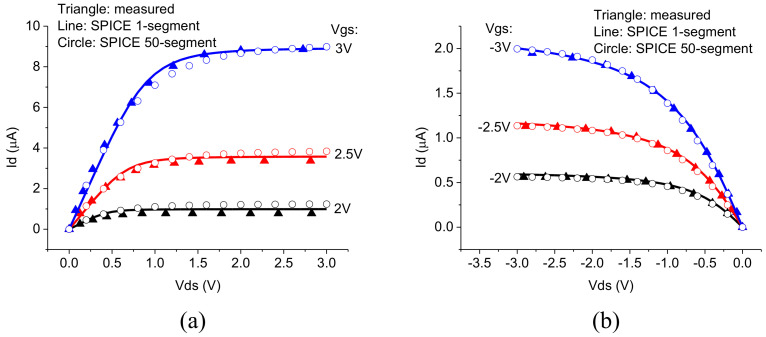
Comparison of the simulated output characteristics for the one segment SPICE model (lines) and the multi-segment SPICE model (circles) with the measured data (triangles) for the 20 μm TFT (**a**) n-channel and (**b**) p-channel.

**Figure 3:**
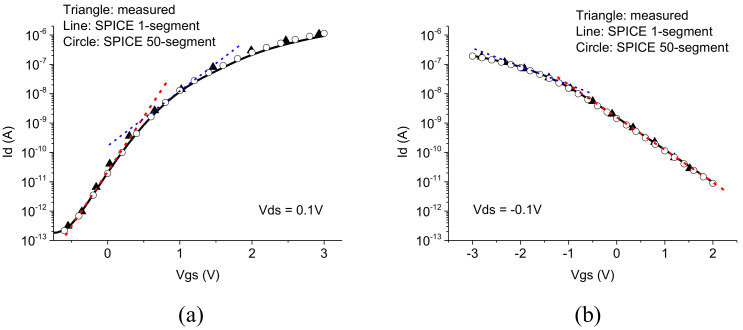
Comparison of the simulated transfer characteristics for the one segment SPICE model (lines) and the multi-segment SPICE model (circles) with the measured data (triangles) for the 20 μm TFT (**a**) n-channel and (**b**) p-channel. The dashed lines show the schematics of the parameter extraction for the two subthreshold slopes.

Figure 2 and Figure 3 show the comparison of the simulated current–voltage characteristics using the TFT-UCCM SPICE model with measured data for n-channel and p-channel TFTs with 20 μm gate length [24]. The multi-segment TFT-UCCM compact model uses 50 segments. Two subthreshold ideality factors describe the control of the channel layer by the gate voltage and the variation of the drain-to-source current and the channel capacitance. The model first calculates the field distribution in the channel, which is then used to evaluate the channel segment parameters including the segment impedance, kinetic inductance, and gate-to-segment capacitances.

Figure 3 illustrates the parameter extraction for the subthreshold slopes by determining the two subthreshold ideality factors on the transition of the subthreshold slopes, which are represented by the two dashed lines. This approach accounts for the physics of the variable channel carrier layer thickness dependence on the gate voltage and allows for accurate fitting of the measured DC characteristics.

**Figure 4:**
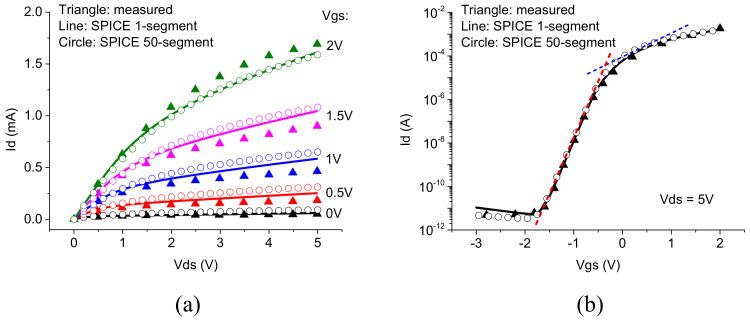
Comparison of the simulated I-Vs for the one segment SPICE model (lines) and the multi-segment SPICE model (circles) with the measured data (triangles) for the 0.8 μm n-channel TFT: (**a**) output characteristics and (**b**) transfer characteristics. The dashed lines in (**b**) illustrates the parameter extraction for the two subthreshold slopes.

Figure 4 illustrates the good agreement of the simulated current–voltage characteristics using the SPICE model with measured data of an n-channel TFT with 0.8 μm gate length [25]. It shows that the model is valid for the simulation of short-channel devices.

### 3.2. Distributed Impedance

**Figure 5:**
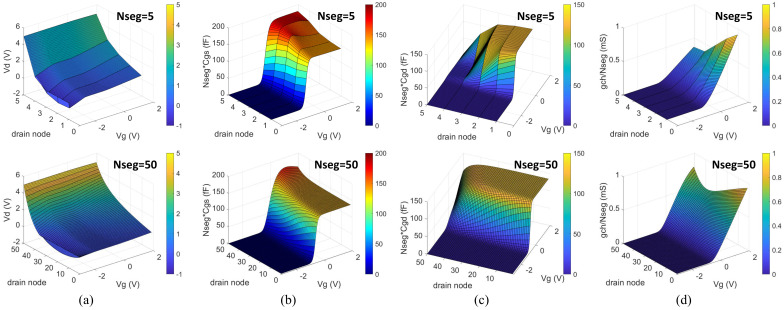
Profiles of the distributed parameters for the TFT-UCCM compact model with 5 segments (Nseg = 5) and 50 segments (Nseg = 50): (**a**) potential at the drain node of each segment in the channel; (**b**) normalized gate-to-source capacitance for each segment; (**c**) normalized gate-to-drain capacitance for each segment; (**d**) normalized channel conductance for each segment.

Figure 5 shows the profiles of the distributed parameters for 5-segments and 50-segments. The simulated results with sweeping the gate bias under a fixed drain bias of 5 V compare the distributed potential at the drain node of each segment in the channel and the distributed nonlinear elements including the gate-to-source and gate-to-drain capacitances for each segment and the segmented channel resistance. It could be seen that more accurate profiles could be achieved by using a greater number of segments.

### 3.3. Cutoff Frequency

**Figure 6:**
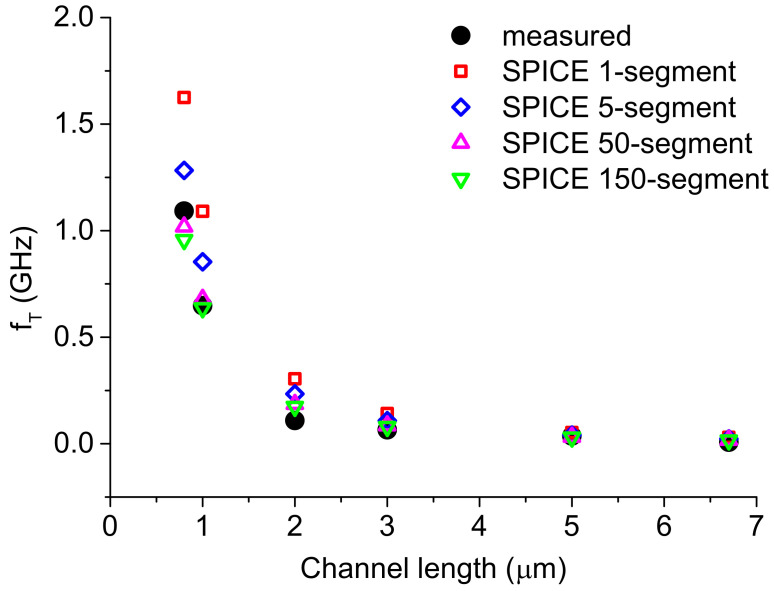
Comparison of the simulated cutoff frequency using the SPICE model of different segments with the measured data. Reprinted with permission from ref. [25]. Copyright 2018 IEEE and Copyright Clearance.

Figure 6 shows the comparison of the simulated cutoff frequency using different segments with the measured results [25]. The model with a larger number of segments shows better agreement in the cutoff frequency with the measured data. This further validates our multi-segment SPICE model for the TFTs.

### 3.4. Application to THz Detection

**Figure 7:**
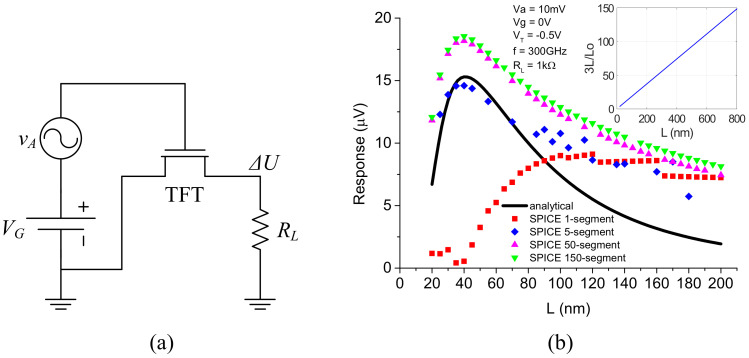
Schematic of a single TFT as a THz detector (**a**) and comparison of the simulated drain response as a function of the gate length with the analytical results (**b**).

FETs can operate in the plasmonic regime well above the device cut-off frequency and have been used to detect THz waves due to the rectification of the nonlinear channel electron density oscillations (plasma waves) induced by the impinging THz radiation [26,27,28]. Figure 7a shows the schematic of using the TFT SPICE model for the simulation of a single TFT as a THz detector. The TFT is biased under asymmetric boundary conditions. $V_{G}$ is the DC gate voltage, $v_{A}$ is the small voltage signal representing the THz radiation, $R_{L}$ is the load resistance, and ΔU is the measurable DC THz response. Figure 7b compares the simulated responses with different segments with the analytical results. The inset figure shows the required number of segments, where $L_{o}$ is the characteristic decay length of the THz ac voltage away from the source [29]. Compared with the 1-segment model, the multi-segment model has a better agreement with the analytical THz response calculated from the THz detection theory [28] and thus should be used for the design and simulation of the TFT based devices and circuits at THz frequencies.

**Figure 8:**
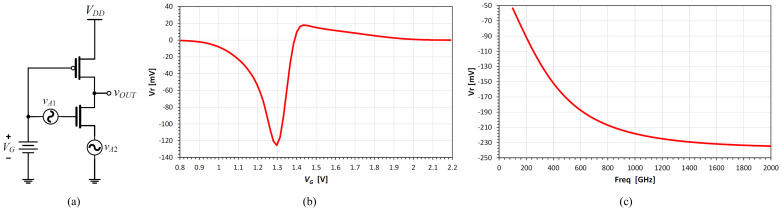
Schematic of a complementary TFT inverter as a THz detector or spectrometer (**a**), simulated output response as a function of the gate bias at a THz radiation frequency of 300 GHz (**b**), and simulated output response as a function of the THz radiation frequency (**c**). The two THz sources have a source impedance of 50 Ω.

Figure 8 shows the application of the multi-segment TFT SPICE model for the simulation of a complementary TFT inverter-based amplifier as a THz detector or spectrometer. Different circuit topologies have been investigated and compared to find out the maximum achievable response [30,31,32]. The improved schematic in Figure 8a shows the best configuration, where the phase-shifted THz input and the complementary technology are used to achieve a high THz response for the TFT technology as illustrated in Figure 8b,c. The predicted THz response with the 0.8 μm n-channel TFT could reach up to hundreds of millivolts.

## 4. Conclusions

The development of the new generation of TFTs (including short channel oxide TFTs) necessitates the development of new compact models that could account for the more sophisticated physics of the electron/hole transport, especially in the subthreshold regime. The presented TFT-UCCM model accounts for the new device physics by introducing double subthreshold slopes and accounting for the distributive capacitance and channel carrier concentration variation with bias. These new features allow us to obtain an excellent agreement with the measured current–voltage and capacitance–voltage characteristics for both long and short n-channel and p-channel TFTs. Even more importantly, the multi-segment approximation extends the model applicability to high frequencies, even beyond the TFT cutoff frequency. In this regime, the TFT non-linearity rectifies the high-frequency signal fed into the TFT and allows detection up to (previously unthought-of) sub-THz and even THz range. We have determined the number of segments that need to be used for the SPICE model to ensure accurate simulation results. Our simulations also show that using the phase-matched feeding the signal into a TFT complementary inverter should yield a very high detection signal; therefore, our result suggests the TFT RFID and other applications could be extended into the THz range of frequencies.

## Data Availability

The data presented in this study are available in this paper.
